# Flashback phenomena after administration of LSD and psilocybin in controlled studies with healthy participants

**DOI:** 10.1007/s00213-022-06066-z

**Published:** 2022-01-25

**Authors:** Felix Müller, Elias Kraus, Friederike Holze, Anna Becker, Laura Ley, Yasmin Schmid, Patrick Vizeli, Matthias E. Liechti, Stefan Borgwardt

**Affiliations:** 1 Department of Psychiatry (UPK), University of Basel, Wilhelm Klein-Strasse 27, 4012 Basel, Switzerland; 2 Division of Clinical Pharmacology and Toxicology, Department of Biomedicine and Department of Clinical Research, University Hospital Basel, University of Basel, 4031 Basel, Switzerland; 3 Department of Psychiatry and Psychotherapy, University of Lübeck, 23538 Lübeck, Germany

**Keywords:** LSD, Hallucinogens, Psychedelics, Psilocybin, Flashbacks, Hallucinogen-persisting perception disorder, HPPD

## Abstract

**Background:**

LSD and psilocybin are increasingly used in phase I trials and evaluated as therapeutic agents for mental disorders. The phenomenon of reoccurring drug-like experiences after the acute substance effects have worn off was described for both substances and especially attributed to LSD. According to the DSM-V, the persisting and distressing manifestation of these experiences is called hallucinogen-persisting perception disorder (HPPD). Data on both conditions is very limited.

**Objective:**

This study aims to provide descriptive data on reoccurring drug-like experiences after the administration of LSD and psilocybin in controlled studies with healthy participants.

**Methods and materials:**

Data from 142 healthy subjects enrolled in six double-blinded, placebo-controlled, randomized cross-over studies were analyzed. In total, 60 subjects received LSD; 27 subjects received LSD, MDMA, and d-amphetamine; 31 subjects received LSD and psilocybin; and 25 subjects received psilocybin and escitalopram. At the end-of-study visit (mean 39.8 days after last study session, SD 37.2), subjects were asked for any reoccurring drug effects since the initial substance effects had worn off. Those reporting reoccurring perception changes more than 24 h after administration were contacted for follow-up (mean follow-up duration: 31.2 months, SD 28.6).

**Results:**

Thirteen out of 142 subjects reported reoccurring drug-like experiences (LSD: seven, psilocybin: two, both: four). The reported phenomena were predominantly mild and perceived as neutral to pleasant. Flashbacks were mostly of visual nature, lasted for seconds to minutes, and occurred within a week after the last drug administration. Two subjects reported distressing experiences that subsided spontaneously. One subject reported brief and pleasant visual perception changes which reoccurred for 7 months. None of the subjects reported impairment in their daily lives. None of the cases met DSM-V criteria for HPPD.

**Conclusion:**

Reoccurring drug-like experiences after the administration of LSD and psilocybin are a common phenomenon occurring in up to 9.2% of healthy subjects (7.8% for LSD, 8.3% for psilocybin and 14.3% if both substances are administered). Additionally, our work suggests that flashback phenomena are not a clinically relevant problem in controlled studies with healthy participants.

## Introduction

In recent years, several studies have investigated effects of LSD and psilocybin in healthy participants (e.g. Griffiths et al. 2006; Schmid et al. 2015) and patients (e.g. Davis et al. 2020; Gasser et al. 2015). These substances are generally regarded as physically well tolerated and non-addictive (Abraham et al. 1996; Johnson et al. 2008; Nichols 2016; Passie et al. 2008). The most important side effects are mental alterations, namely induction of flashbacks or psychosis (Nichols 2016; Passie et al. 2008).

According to the International Classification of Diseases (ICD-10, F16.7, WHO 2010), flashbacks are defined as episodic recurrences of drug effects after the acute pharmacological effects have subsided and are characterized as mostly very transient.

In clinical settings, recurrent drug effects after intake of hallucinogens have been reported in a study on the use of LSD in psychiatric treatments as early as the 1950s (Sandison et al. 1954). Although these phenomena are therefore known for decades, they are still poorly described with little consensus on their cause (Halpern and Pope 2003; Hermle et al. 2008, 2015; Holland and Passie 2011; Lerner et al. 2014b; Martinotti et al. 2018). Reported incidences vary widely, with estimates between 15 and 75% of all users being affected (e.g. Hermle et al. 2015; Stanton and Bardoni 1972). Most cases were reported after the use of LSD (Halpern and Pope 2003; Lerner et al. 2014b), but flashbacks were also seen after the use of MDMA (Vizeli and Liechti 2017) as well as cannabis (Lerner et al. 2011), amphetamines, tobacco, and alcohol (Lerner et al. 2014b) and rarely after psilocybin (Hermle et al. 2015). Most commonly reported symptoms are changes in vision, a great variety of which have been reported, i.e. afterimages, geometrical patterns, and intensified perception of colours (Abraham and David Abraham 1983; Hermle et al. 2008; Lerner et al. 2014b). Other phenomena comprise changes in mood/affect and derealization/depersonalization (Lerner et al. 2014b). Reports of new symptoms—which were not initially experienced during the acute drug effects—exist, but seem to be rare (Lerner et al. 2014a). Awareness of the illusory nature of the phenomenon is usually present (Hermle et al. 2008; Lerner et al. 2014b).

According to the Diagnostic and Statistical Manual of Mental Disorders (DSM-V, American Psychiatric Association 2013), persisting flashback phenomena which cause clinically significant distress or impairment can be classified as hallucinogenic persisting perception disorder (HPPD, DSM-V, 292.89). Another classification differentiates between type 1 and type 2 HPPD (Halpern et al. 2018). Type 1 HPPD is consistent with the ICD-10 definition and is characterized by transient recurrences of alterations in perception, mood, or consciousness as experienced during the acute drug effects. These alterations might be perceived as pleasant. In contrast with this, type 2 HPPD is characterized by visual phenomena which are constantly or almost constantly present. These symptoms are mostly experienced as distressing and often accompanied by depersonalization, derealization, anxiety, or depression.

Available descriptions of HPPD stem mostly from case reports of hallucinogen users seeking medical help because of significant distress and impairment of daily life due to the persisting perception changes (Halpern and Pope 2003; Lerner et al. 2014b). In most presented cases, the exact circumstances, involved substances, and dosages are unknown or rely on patient memory (Halpern and Pope 2003; Martinotti et al. 2018; Orsolini et al. 2017). Courses seem to vary widely with onsets from hours to years after drug use, episodes that last from seconds to years, and severity ranging from mild to severe and debilitating (Abraham et al. 1996; Lerner et al. 2014b; Martinotti et al. 2018). Pre-existing psychiatric conditions such as major depression, bipolar disorder, schizophrenia spectrum disorders, polysubstance use (Halpern and Pope 2003; Lerner et al. 2014b; Martinotti et al. 2018) or tinnitus, and migraine (Puledda et al. 2020) are proposed risk factors. However, the condition can arise in healthy individuals after a single hallucinogen use (Abraham et al. 1996; Martinotti et al. 2018). Reported incidences vary widely. A systematic review of the literature concluded that HPPD seems to be rare but that an estimate of the incidence is not possible due to insufficient data (Halpern and Pope 2003). In contrast, the DSM-V reports a prevalence of 4.2% among users of hallucinogens.

Overall, current knowledge with regard to flashback phenomena and HPPD is very limited and mostly based on case reports and naturalistic studies. However, flashbacks are assumed to be among the most relevant side effects of hallucinogenic drugs. Given the renewed scientific interest to use these compounds in clinical trials and as potential therapeutic agents (Müller et al. 2020), these phenomena should be investigated more carefully. Specifically, we are not aware of a report on flashback phenomena in modern controlled LSD studies. To our knowledge, there is only one report on a pooled data set of controlled clinical studies that administered a hallucinogenic drug (Studerus et al. 2011). The authors investigated the occurrence of altered state of consciousness before and after the administration of psilocybin in 110 healthy participants. Interestingly, the incidence of these events was ~ 10% before and after the study sessions, which questions the induction by the study drug. Therefore, this study aims to quantitatively and qualitatively describe flashback phenomena and HPPD in a pooled data set of several clinical trials in healthy participants. In total, this analysis covers data from 142 healthy subjects enrolled in six clinical trials. Of these, 60 subjects received LSD; 27 subjects received LSD, MDMA, and d-amphetamine; 31 subjects received LSD and psilocybin; and 25 subjects received psilocybin and escitalopram.

## Methods

### Studies included in analysis

This analysis is based on the pooled data sets of all available clinical trials involving LSD or psilocybin conducted by our team in Basel (Switzerland) as of April 2021. The Ethics Committee for Northwest/Central Switzerland (EKNZ) and the Federal Office of Public Health approved all trials. Studies were conducted in accordance with the Declaration of Helsinki. Informed consent was obtained from all participants.

All studies were double-blinded, placebo-controlled, randomized, cross-over trials in healthy participants. The overall sample comprised 142 participants enrolled in six studies. In total, 90 participants received LSD, 24 participants received psilocybin, and 28 participants received both substances. Two studies (*n* = 16 and 24, respectively) tested the effects of a single administration of 0.1 mg and 0.2 mg LSD, respectively (Dolder et al. 2016; Schmid et al. 2015). One study (*n* = 27) compared 0.1 mg LSD with d-amphetamine and MDMA (Holze et al. 2020). Another study (*n* = 20) compared different doses of LSD (0.025 to 0.2 mg) and LSD in combination with ketanserin (Holze et al. 2021). Another study (*n* = 24) tested the effects of 25 mg psilocybin after pre-treatment with escitalopram or placebo (Becker et al. 2021). Lastly, effects of 0.1 and 0.2 mg LSD were compared with effects of 15 and 30 mg psilocybin (*n* = 31) by the sixth study (not yet published; NCT03604744). Three participants of this study dropped out after the administration of LSD and did not receive psilocybin. Overall, LSD was administered 213 times (1–5 times per person in doses ranging from 0.025 to 0.2 mg), and psilocybin was administered 103 times (1–2 times per person in doses ranging from 15 to 30 mg). LSD was prepared as gelatine capsules (Dolder et al. 2016; Schmid et al. 2015) or as solution in alcohol (units of 0.025 or 0.1 mg in 1 mL of ethanol; Holze et al. 2020, 2021, NCT 03604744) and administered orally. Psilocybin was prepared as gelatine capsules (units of 5 mg) and administered orally (NCT03604744, (Becker et al. 2021)).

### Inclusion and exclusion criteria of studies

To qualify for participation in any of the studies, the following inclusion criteria were applied: age between 25 and 65 years (for NCT03019822: 25–50 years), sufficient understanding of the German language, understanding of the procedures and risks associated with the study, willingness to adhere to the protocol, informed consent, refraining from taking illicit psychoactive substances during the study, willing to use an effective form of birth control, and abstaining from alcohol or xanthine containing liquids or food on the evening before study sessions and on the study day itself. Exclusion criteria comprised personal or first-grade relative history of seizures, cardiac or neurological disorders, current or previous psychotic or major psychiatric disorder, history of psychotic disorder in first degree relatives, relevant prior illicit drug use more than ten times (except THC-containing products) or any illicit drug use within the previous 2 months, pregnancy or nursing women, and use of medication that may interfere with the effects of the study substances. Some of the studies (NCT03604744, NCT03019822, NCT02308969, NCT03321136, NCT03912974) applied additional criteria (i.e. additional specific medical conditions, fMRI-related criteria). Details of all six trials are shown in Table 1.

**Table 1:** Study and demographic characteristics

Study	Substance	Doses	Subjects	Gender	Age (years)	Previous cannabis use	Previous hallucinogen use			Previous stimulant use			Previous MDMA use			No. of subjects with flashbacks		Interval between last session and end-of-study visit (days)		Interval between last session and follow-up (days)	
NCT identifier	Name	mg	*n*	f/m	Mean (SD)	%	%	Mean (SD)	Range	%	Mean (SD)	Range	%	Mean (SD)	Range	*n*	%	Mean (SD)	Range	Mean (SD)	Range
NCT02308969	LSD	0.1	24	12/12	32.5 (11.1)	75.0	16.7	0.2 (0.5)	0–2	16.7	0.3 (0.7)	0–2	33.3	0.9 (1.6)	0–5	1	4.2	27.6 (24.1)	8–112	Lost to follow-up	Lost to follow-up
NCT03019822	LSD, MDMA, amph	0.1, 125, 40	27	14/13	28.0 (4.2)	85.2	11.1	0.1 (0.5)	0–2	44.4	0.9 (1.3)	0–4	29.6	0.8 (1.5)	0–5	2	7.4	87.6 (45.6)	35–193	1117.0	1082–1152
NCT03321136	LSD	0.025, 0.05, 0.1, 0.2	20	11/9	29.3 (6.3)	85.0	40.0	0.7 (1.0)	0–3	55.0	1.1. (1.3)	0–3	50.0	1.2 (1.6)	0–5	2	10.0	31.5 (21.8)	6–93	969.0	828–1110
NCT01878942	LSD	0.2	16	8/8	28.7 (6.4)	87.5	43.8	0.8 (1.0)	0–3	37.5	0.7 (1.1)	0–3	62.5	1.7 (2.0)	0–8	2	12.5	26.1 (9.8)	4–44	2644.5	2641–2648
NCT03604744	LSD, psilo	0.1 and 0.2, 15 and 30	31	17/14	36.1 (10.3)	90.3	45.2	1.3 (1.9)	0–8	41.9	0.9 (1.4)	0–5	41.9	1.1 (1.6)	0–5	4	12.9	40.5 (30.4)	6–140	382.5	101–589
NCT03912974	Psilo, escitalopram	25, 20	24	12/12	33.7 (9.7)	50.0	29.2	0.7 (1.5)	0–5	12.5	0.2 (0.7)	0–3	33.3	0.8 (1.4)	0–5	2	8.3	13.6 (12.0)	2–56	216.0	158–274
Total			142	74/68	31.7 (9.0)	78.9	30.3	0.7 (1.3)	0–8	34.5	0.7 (1.2)	0–5	40.1	1 (1.6)	0–8	13	9.2	39.8 (37.2)	2–193	951.9 (871.1)	101–2648

*amph* d-amphetamine, *f* female, *m* male, *NCT* National Clinical Trial number, *psilo* psilocybin, *SD* standard deviation

### Data collection

As part of the routine study procedure, subjects were asked at each study session to report any adverse events since the last contact with the study team. Any event, including flashbacks, was recorded. Additionally, all studies included an end-of-study visit after the last study session. At this visit, all subjects were asked for the occurrence of flashback phenomena over the whole course of the respective study. If subjects reported flashbacks, they were asked to describe the phenomenon with regard to quality, quantity, impairment, and time of occurrence.

### Follow-up

Subjects reporting a flashback at their end-of-study visit were contacted via e-mail for a follow-up in May 2021 in order to assess the occurrence of further flashbacks or HPPD. If they reported further flashbacks, they were asked to describe them with regard to quality, quantity, frequency, and impairment. Subjects were also asked if they are able to identify triggers of their flashbacks.

### Hallucinogen-persisting perception disorder

Based on this data collection, it was evaluated whether subjects met DSM-V criteria for HPPD at any time point after administration of the study drugs. As HPPD is considered to be relatively rare (Halpern and Pope 2003), we also tested the probability of observing zero cases in our sample of 142 subjects. Because the exact incidence of HPPD is unknown, different frequencies were tested. To this end, 100,000 equally sized samples at different assumed incidences (1%, 2%, 3%, 4%, and 5%) were simulated. The probability of observing zero cases was calculated for each incidence. The R function rbinom (R Version 4.0.5) was used for this simulation (Fox 2016; R Core Team 2021; Schauberger and Walker 2020).

## Results

### Subject characteristics

One hundred forty-six participants were initially enrolled. Due to dropouts before administration of LSD or psilocybin, the final sample of this analysis comprised 142 subjects (74 female, 68 male) with a mean age of 31.7 years (SD 9.0). Overall, hallucinogens had been used prior to the study by 30.3% of the participants on an average of 2.2 occasions (SD 1.4). MDMA had been used by 40.1% on 2.6 occasions (SD 1.6). Other stimulants had been used by 34.5% on 2.0 occasions (SD 1.1). Most of the participants had prior use of cannabis (78.9%). Use of other illicit substances was rare (dissociatives: seven subjects, opioids: two subjects, sedatives: one subject). Complementary details for each study are shown in Table 1.

### Flashback phenomena at end-of-study visit

The interval between the last sessions and the end-of-study visit was 39.8 days on average (SD 37.2, range 2 to 193). At this visit, 13 subjects (mean age 31.4 years, SD 9.2) reported flashback phenomena of some sort (corresponding to 9.2% of all participants). Ten of them were female (76.9%) and three (23.1%) were male. Seven subjects experienced flashbacks after the administration of LSD (7.8%), two after the administration of psilocybin (8.3%), and four after the administration of both substances (14.3%). In all but two cases, the occurrence of flashbacks was limited to the week after the last drug administration. Eleven participants (84.6%) reported visual alterations, which were accompanied by other phenomena (auditory, cognitive, feeling of disintegration) in three cases (23.1%). Two subjects (15.4%) exclusively reported emotional alterations. In almost all cases, phenomena lasted for seconds (69.2%) to minutes (23.1%). In one case (7.7%), alterations persisted for hours. This subject reported intensified perception of colours and slowed thinking on the respective day after three study sessions. In 53.8% of the cases, the phenomena only occurred once. In two cases (15.4%), symptoms reoccurred more than five times. One of these subjects experienced around 20 visual flashbacks within a short period approximately 24 h after drug administration. The other subject experienced approximately 30 visual flashbacks within a period of 7 months after drug administration. At follow-up, this was the only participant who clearly reported reoccurrence of flashbacks after the end-of-study visit (see below). In both cases, flashbacks lasted for seconds, were experienced as benign, and did not impair daily life. Over 50% of the subjects reported that the phenomena occurred during relaxing or shortly before or after sleeping. Please see Table 2 for complementary details.

**Table 2:** Characteristics of flashback phenomena

Study	Subject	Gender	Age	Administration after which flashback occurred	Other administrations before onset of flashback	Domain	Number of flashbacks	Description of phenomena	Duration	Onset after administration	Last occurrence after administration	Circumstances	Interpretation	Impairment of daily life	Reoccurrence at follow-up
NCT02308969	1	f	28	0.1 mg LSD	-	Visual, auditory	2	Visual pseudohallucinations, changed perception of music	5 min	2 days	-	Listening to music	Neutral	No	Unclear (1)
NCT03019822	2	m	27	0.1 mg LSD	40 mg amph	Visual	1	Visual pseudohallucinations	Secs	~ 7 days	-	Waking up	Positive	No	No
	3	m	25	0.1 mg LSD	40 mg amph, 125 mg MDMA	Emotional	1	Positive mood	1 min	1 day	-	Listening to music	Positive	No	No
NCT03321136	4	f	40	0.2 mg LSD	0.05 mg LSD	Visual, self-structure	4	Visual pseudohallucinations, feeling of disintegration of the self	Secs	1.5 days	4.5 days	Falling asleep	Distressing	No	No
	5	f	30	0.05 mg, 0.1 mg, 0.2 mg LSD	25 mcg LSD	Visual, cognitive	3	Intensified perception of colours, slowed thinking	Hours	1 day	-	No specific	Neutral	No	No
NCT01878942	6	f	27	0.2 mg LSD	-	Visual	1	Intensified perception of colours	Secs	3.5 days	-	Unclear	Unclear	No	No
	7	f	52	0.2 mg LSD	-	Visual	1	3D perception of a 2D object	Secs	1.5 days	-	Reading	Positive	No	No
NCT03912974	8	f	26	25 mg psilo	-	Visual	1	Visual pseudohallucinations	Secs	2.5 days	-	Dinner	Neutral	No	No
	9	f	25	25 mg psilo	2 × 10 mg escitalopram	Visual	1	Altered perception of light	Secs	17 days	-	Falling asleep	Distressing	No	Unclear (1)
NCT03604744	10	f	25	30 mg psilo, 0.2 mg LSD	15 mg psilo	Emotional	4	Euphoric mood	Secs	2 days	6 days	Waking up	Positive	No	No
	11	m	29	0.1 mg LSD	0.2 mg LSD, 15 mg psilo	Visual	1	Visual pseudohallucinations	1 min	2 days	-	Relaxing	Neutral	No	No
	12	f	26	0.2 mg LSD	30 mg psilo, 15 mg psilo, 0.1 mg LSD	Visual	~ 30	Visual pseudohallucinations	Secs	1 day	7 months	No specific	Positive	No	Yes
	13	f	48	30 mg psilo	0.1 mg LSD	Visual	~ 20	Visual illusion	Secs	1 day	-	No specific	Neutral	No	No

(1): please see results. *amph* d-amphetamine, *f* female, *m* male, *psilo* psilocybin, *min* minutes, *secs* seconds

Flashbacks were experienced as unpleasant in two cases (15.4%) and as neutral or positive in ten cases (76.9%). One case was not sufficiently documented. Overall, 1.4% of all 142 subjects participating in our trials reported distressing experiences related to flashbacks. None of the subjects reported impairment of daily life due to these symptoms. One of these participants experienced visual alterations and disintegration of the self for a few seconds on four consecutive days while falling asleep. It is noteworthy that the study session after which these flashbacks occurred had been exceptionally difficult. After administration of 0.2 mg LSD, the subject experienced nausea and disintegration of the self and vomited repeatedly for several hours. She dropped out of the study after this session. At follow-up after 36 months, no flashbacks had reoccurred. The other participant described her study session after the administration of 25 mg psilocybin as “pleasant and uncomplicated”. A single flashback occurred 17 days after this session, after two administrations of 10 mg escitalopram daily that were part of the pre-treatment for her second study session. She reported an altered perception of light while falling asleep after a stressful day. This altered perception caused considerable anxiety as she catastrophically interpreted the incident as a sign of emerging substance-induced psychosis. Later on, she attributed the incident to the ingestion of escitalopram rather than psilocybin. While there were no further visual alterations after the described event, the subject developed symptoms typical of panic attacks and anticipatory anxiety with regard to these attacks. Finally, she dropped out of the study because of these events. It is possible that the experience and more specifically the interpretation of the flashback promoted the occurrence of these attacks. The subsequent panic attacks were accompanied by several symptoms that could be seen as further flashbacks (e.g. feelings of derealization); however, the combination of symptoms was more typical for panic attacks. For that reason, we do not interpret the episodes that occurred after the first visual episode as further flashback phenomena.

### Flashback phenomena at follow-up

On average, the follow-up took place 31.2 months after the end-of-study visit. Due to different completion dates of the included studies, time to follow-up varied considerably (SD 28.6, range 3.3 to 86.8 months). All but one of the subject responded to our follow-up mail (response rate ~ 92%). One subject was unreachable. Another subject did not provide all requested information and was unreachable afterwards.

One subject reported reoccurrence of flashback phenomena after the end-of-study visit, while all other subjects (92.3%) did not reexperience any flashbacks. The subject in question reported that she had experienced approximately 30 flashbacks over a period of 7 months. Flashbacks were of the same visual nature (“seeing of glowing dots”) as reported at the end-of-study visit. They lasted for seconds, were experienced as benign, and did not impair daily life. At the time of follow-up, these visual alterations had ceased approximately 10 months ago.

### Hallucinogen-persisting perception disorder

None of the 142 subjects in our sample met DSM-V criteria for HPPD at any time during our investigation. The calculated probabilities of observing zero cases of HPPD in a sample of 142 subjects were 24.0% for an assumed true incidence of 1%; 5.7% for an assumed 2% incidence; and 1.3% for 3%, 0.3% for 4%, and 0.1% for an assumed 5% incidence respectively.

## Discussion

This work analyzed pooled data from six trials investigating the effects of LSD, psilocybin, MDMA, amphetamine, and escitalopram in healthy participants. The presented data suggests that flashback phenomena after administration of LSD and psilocybin in healthy participants are relatively common in clinical trials, affecting approximately 9% of the subjects. However, it should be mentioned that three of the reported cases also received non-hallucinogenic substances (escitalopram, d-amphetamine, MDMA) which might modulate (Halpern et al. 2018; Markel et al. 1994) or perhaps induce flashback phenomena (Lerner et al. 2014b; Vizeli and Liechti 2017). The frequency observed in our sample was lower than reported in several naturalistic studies, where incidences of 15 to 75% were seen (Hermle et al. 2015). In line with the description provided by the ICD-10, flashbacks were of short duration, transient, and mostly of visual nature. They were mostly experienced as benign. Cases of three participants seem to be most relevant: one of these subjects experienced repeated, brief flashbacks over a period of several months. This phenomenon started 1 day after the administration of 0.2 mg LSD, but the subject had also received psilocybin (15 and 30 mg) and a lower dose of LSD (0.1 mg) before onset. The phenomenon was experienced as benign, did not impair daily life, and ceased spontaneously. Two other subjects experienced transient flashbacks which were experienced as distressing. One of these subjects experienced a single, distressing episode 17 days after the administration of 25 mg psilocybin, at the second day of an administration of a serotonin reuptake inhibitor. This is in line with three cases reported elsewhere, where serotonin reuptake inhibitors induced or worsened flashbacks in users of hallucinogenic drugs (Halpern et al. 2018; Markel et al. 1994). Notably, this subject also had the longest interval between administration of the hallucinogenic study drug and the occurrence of the flashback. The other subject experienced distressing flashbacks on four consecutive days directly after the administration of 0.2 mg LSD (and prior administration of 0.05 mg LSD at another occasion). Both subjects were not impaired in their daily lives, and the condition was resolved spontaneously within a week of drug administration.

In contrast to existing literature, where flashback phenomena are more commonly reported after the use of LSD compared to psilocybin (Holland and Passie 2011), incidences in our sample were comparable (7.8% for LSD and 8.3% for psilocybin). However, it should be noted that data for psilocybin is based on a small sample of 24 subjects. In a pooled analysis of controlled studies with psilocybin in 110 healthy participants, Studerus et al. found that ~ 10% of the participants reported spontaneous altered states of consciousness before and after the administration of psilocybin (Studerus et al. 2011). Notably, the definition of altered states of consciousness applied by Studerus et al. was very broad and included states such as lucid dreams. Only three participants reported visual alterations and it was not specified whether these experiences occurred before or after exposure to the study drug and are therefore not classifiable as flashbacks per se. In this context, it is also interesting to note that we have observed a comparable incidence of flashback phenomena in a pooled analysis of studies with MDMA in healthy participants. In a sample of 131 subjects, 9% reported flashbacks of some sort, which occurred within 8 to 50 h after drug administration (Vizeli and Liechti 2017). Only taking into account incidences > 24 h after administration (in line with the definition of this analysis), flashbacks were reported by 6.5% of the participants. This might indicate that subjects in trials on psychoactive substances commonly report flashback-like experiences.

The etiology of flashback phenomena is unknown. According to a popular hypothesis, flashbacks are due to substance-induced, chronic dysfunction of inhibitory neurons resulting in disinhibition of visual processes (Abraham and Duffy 1996; Martinotti et al. 2018). Another hypothesis states that flashbacks are involuntary memories shaped by the unusually distinct experience induced by hallucinogenic drugs (Halpern and Pope 2003; McGee 1984; Shick and Smith 1970). However, it is also possible that subjects in studies with psychoactive drugs are more receptive for phenomena which are at the borderline between normal and abnormal experiences (Halpern and Pope 2003). In fact, it has been noticed that visual alterations and related phenomena are not uncommon in healthy persons (McCreery and Claridge 1996; Yung et al. 2009). Therefore, several of the reported experiences (e.g. intensified perception of colours or music) could also be explained by heightened awareness with regard to such phenomena. Over 50% of the participants in this study reported that flashbacks occurred before sleeping, after waking up, or during relaxing. This might indicate that awareness for such experiences was heightened when they were not engaged in activities. Alternatively, these circumstances might be cues for flashbacks (McGee 1984) as there might be some similarities to the experiences during the study sessions.

Some of our cases met the ICD-10 definition for flashbacks (F16.7). This definition requires the episodic recurrences of drug-like experiences. If “episodic” is interpreted as occurrence on more than one occasion, this definition is met in six cases. In contrast, none of our cases meets DSM-V criteria for HPPD. This is line with investigations during the 1960s and 1970s, where no case of HPPD was seen in a sample of several thousand persons who received LSD in clinical settings (Cohen 1960; Hermle et al. 2015; Malleson 1971). More recently, a similar result was reported for several trials on psilocybin in healthy subjects (Studerus et al. 2011). According to the DSM-V, HPPD has a prevalence of 4.2% among recreational users (American Psychiatric Association 2013). According to our simulations, our finding of observing zero cases of HPPD would have a < 1.3% chance of occurring, should the true incidence of HPPD be higher than 3%. Therefore, it is unlikely that this observation was due to chance. It should be noted that estimates of the incidence of HPPD vary relatively widely. Therefore, the true incidence might be lower and decreases the probability to observe any cases in the current analysis. However, our result might also indicate that cases of HPPD are more unlikely to occur in clinical trials. As there is no consensus with regard to risk factors for HPPD, we can only speculate about possible causes. Some findings suggest that mental disorders like major depression, bipolar disorder, psychosis, and polysubstance—which were exclusion criteria in our trials—are associated with HPPD (Halpern and Pope 2003; Lerner et al. 2014b; Martinotti et al. 2018). Others suggest that HPPD is related to the number of exposures to the drug (Abraham and David Abraham 1983; McGlothlin and Arnold 1971) which was relatively low in our sample.

This work has several other limitations. Firstly, it is limited by the relatively small sample size of 142 subjects. As noted, this might be a particular problem for the assessment of HPPD, which is considered to be a rare condition. In addition, doses and administered substances varied across studies. For that reason, it was not possible to determine which dose or substance might have caused the flashback phenomena in some cases. Furthermore, it is likely that participants with positive attitudes towards hallucinogenic drugs or positive previous experiences were enrolled in these studies, which may result in a lower rate of negatively experienced events. Likewise, a substantial part of the participants had used other illicit drugs (especially cannabis) before enrolment. It is possible that individuals who have already experienced negative after-effects are less likely to participate in such studies. Moreover, we only followed up participants who reported flashbacks at the end-of-study visit and flashbacks may have occurred in other subjects later on. Furthermore, the follow-up occurred at different timespans due to different dates of study completion. Lastly, subjects were screened using various criteria, among others the absence of mental diseases. The occurrence of flashback phenomena and HPPD in patients might be different.

## Conclusion

Drug-like experiences after the administration of LSD and psilocybin seem to be a relatively common phenomenon in clinical trials with healthy participants. However, the flashback phenomena observed in this study were transient, mostly experienced as benign and did not impair daily life. Overall, 1.4% of the subjects participating in our trials reported distressing experiences related to flashback phenomena and these conditions did not require treatment. No cases of HPPD according to DSM-V criteria occurred in our sample. Overall, our data suggests that flashbacks are not a clinically relevant problem in controlled studies with healthy participants.
